# Cortical fractal dimension predicts disability worsening in Multiple Sclerosis patients

**DOI:** 10.1016/j.nicl.2021.102653

**Published:** 2021-03-29

**Authors:** Eloy Roura, Grégory Maclair, Magí Andorrà, Ferran Juanals, Irene Pulido-Valdeolivas, Albert Saiz, Yolanda Blanco, Maria Sepulveda, Sara Llufriu, Eloy Martínez-Heras, Elisabeth Solana, Elena H Martinez-Lapiscina, Pablo Villoslada

**Affiliations:** a Health Engineering SL, Getxo, Spain; b Institut d’Investigacions Biomèdiques August Pi Sunyer – Hospital Clinic, University of Barcelona, Spain; c Stanford University, Stanford, CA, USA

**Keywords:** Multiple Sclerosis, Disability progression, Clinical outcomes, Brain MRI, Fractal dimension

## Abstract

**Background:**

Fractal geometry measures the morphology of the brain and detects CNS damage. We aimed to assess the longitudinal changes on brain’s fractal geometry and its predictive value for disease worsening in patients with Multiple Sclerosis (MS).

**Methods:**

We prospectively analyzed 146 consecutive patients with relapsing-remitting MS with up to 5 years of clinical and brain MRI (3 T) assessments. The fractal dimension and lacunarity were calculated for brain regions using box-counting methods. Longitudinal changes were analyzed in mixed-effect models and the risk of disability accumulation were assessed using Cox Proportional Hazard regression analysis.

**Results:**

There was a significant decrease in the fractal dimension and increases of lacunarity in different brain regions over the 5-year follow-up. Lower cortical fractal dimension increased the risk of disability accumulation for the Expanded Disability Status Scale [HR 0.9734, CI 0.8420–0.9125; Harrell C 0.59; Wald p 0.038], 9-hole peg test [HR 0.9734, CI 0.8420–0.9125; Harrell C 0.59; Wald p 0.0083], 2.5% low contrast vision [HR 0.4311, CI 0.2035–0.9133; Harrell C 0.58; Wald p 0.0403], symbol digit modality test [HR 2.215, CI 1.043–4.705; Harrell C 0.65; Wald p 0.0384] and MS Functional Composite-4 [HR 0.55, CI 0.317–0.955; Harrell C 0.59; Wald p 0.0029].

**Conclusions:**

Fractal geometry analysis of brain MRI identified patients at risk of increasing their disability in the next five years.

## Highlights

• Fractal geometry measures the morphology and damage of the brain in MS.
• Prospective study with up to 5 years of clinical and MRI assessments.
• Brain fractal dimension decreases and lacunarity increases over the 5-years.
• Cortical fractal dimension identified patients at risk of disability worsening.
• Fractal geometry analysis complement MRI monitoring of MS progression.

## Graphical abstract

**Figure:**
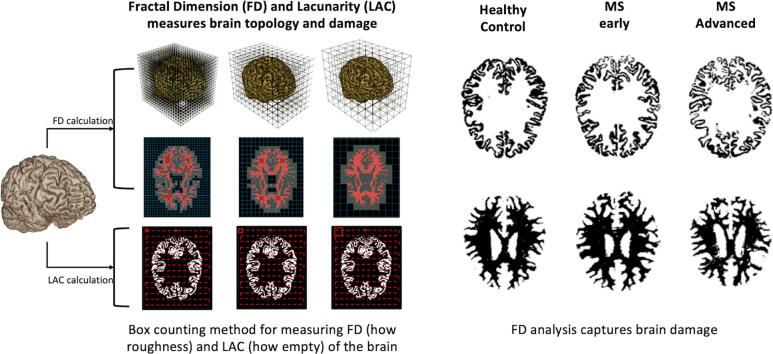

## 1 Introduction

The course of Multiple Sclerosis (MS) is highly unpredictable, but tissue damage accumulates overtime at different speeds, creating significant clinical heterogeneity (Thompson et al., 2018, Kotelnikova et al., 2017). Due to the high uncertainty regarding the disease course in each patient, prognostic biomarkers are being pursued to support the decision-making process and to employ precision medicine when defining the best therapeutic regimen for a given patient (Pulido-Valdeolivas et al., 2017). Brain magnetic resonance imaging (MRI) is the most informative and well-studied prognostic biomarker, having been included in the diagnostic criteria (Polman et al., 2010) and in the recommendations to assess the response to therapy as a predictive biomarker (Wattjes et al., 2015).

The fractal dimension of the brain is altered significantly in individuals with MS, both that corresponding to the gray and the white matter obtained from either 2D or 3D MRI images (Esteban et al., 2007, Esteban et al., 2009). Indeed, similar alterations to the fractal dimension of the brain have also been found in other CNS diseases like dementia, stroke, or conditions like prematurity (Esteban et al., 2010, Di Ieva et al., 2015). The brain shows such fractal properties over certain scales, and hence, analyzing the fractal geometry (fractal dimension or lacunarity) of the brain reflects abnormalities due to tissue damage (Di Ieva et al., 2014). Changes in the fractal dimension are associated with the presence of focal lesions or microscopic alterations to the shape of the grey and white matter (Di Ieva et al., 2015). The higher the fractal dimension, and the lower the lacunarity, the more complex and healthier is the brain. For this reason, fractal geometry is altered as brain damage increases, and it is therefore associated with corresponding disability.

Because the fractal dimension of the brain is calculated from T1 sequences, it is a metric that can be readily applied in the clinic without the need for a specialized scanner or sequences. Indeed, the brain’s fractal dimension is little influenced by variability in the scanner and sequences, becoming less noisy than other MRI metrics (Di Ieva et al., 2015, Krohn et al., 2019). For this reason, the fractal dimension analysis of brain MRI is being pursued as a potential biomarker to monitor brain damage.

The objective of this study was to assess the changes in fractal geometry of the brain in people with MS and to assess whether changes in fractal dimension predicts the changes in disability accumulation in the short to medium term (up to 5 years).

## 2 Methods

### 2.1 Patients

We recruited the first 146 consecutive relapsing-remitting MS patients fulfilling the criteria approved at the time of inclusion (2010 McDonald criteria (Polman et al., 2010) of the prospective MSVisualPath cohort, followed at the Hospital Clinic of the University of Barcelona (Spain), which started in 2011 and is described in detail elsewhere (Martínez-Lapiscina et al., 2014, Andorra et al., 2018). We also collected 34 healthy volunteers for baseline comparisons. Patients were allowed to change disease-modifying therapies as indicated previously (Andorra et al., 2018), and they were subjected to a baseline and annual clinical and brain MRI assessment over 5 years, except for year 4. Thus, five assessments were made on each patient. The study was approved by the Ethical Committee of the Hospital Clinic of Barcelona, and the patients were recruited by their neurologist after providing their signed informed consent.

### 2.2 Clinical assessments

The variables collected included the Expanded Disability Status Scale (EDSS) (Kurtzke, 1983), MS Severity scale (MSSS) (Roxburgh et al., 2005), Age-Related MSSS (ARMSS) (Manouchehrinia et al., 2017), the MS functional composite 4 (MSFC-4) (Goldman et al., 2019), Timed 25 feet Walking Test (T25WT) (Motl et al., 2017), 9-Hole Peg Test (9HPT) (Feys et al., 2017), Symbol Digit Modality Test (SDMT) (Strober et al., 2018), low contrast visual acuity using the Sloan 2.5% contrast cards (SL25) (Balcer et al., 2017), and No Evidence of Disease Activity (NEDA, defined as no relapses, no increase on the EDSS, and no new Gad+ or T2 lesions) (Giovannoni et al., 2018). At the time of analysis, the number of cases that either has not achieved a 5-year follow-up or for which there was missing data regarding the different clinical scales were: EDSS: 0; 9HPT: 24; T25WT: 29; SDMT: 8; and SL25: 9. The EDSS and the other outcomes were confirmed at each yearly visit based on the results of the clinical visit 6-months earlier to define the Confirmed Disability Accumulation (CDA). The EDSS related CDA was established as an increase of 1 point on the EDSS (for an EDSS at baseline between 0 and 5.5) or of 0.5 points for patients with an EDSS at baseline ≥ 5.5 confirmed after 6 months. For the 9HPT and T25WT, the CDA was defined as a 20% change in each score, whereas it was 4 points for the SDMT and 7 letters for the SL25 confirmed after 6 months as described in (Goldman et al., 2019).

### 2.3 Brain MRI acquisition and image processing

The MRI acquisition and analysis carried out on this cohort is described in detail in (Andorra et al., 2018). Briefly, MRI studies were performed with a 3 T Magnetom Trio scanner (Siemens). The scans were acquired using a 32-channel phased-array head coil. In this study, we used the 3-dimensional (3D) structural T1-weighted voxel magnetization-prepared rapid gradient echo (T1-MPRAGE, voxel size 0.9 × 0.9 × 0.9 mm^3^), 3-D T2-fluid-attenuated inversion recovery (T2-FLAIR) images with the same voxel size to quantify the change in brain volume. In short, the T2-FLAIR images were registered to the T1-MPRAGE scans to ease the manual segmentation of the lesions by a trained neurologist. Moreover, post-gadolinium T1 (gradient-echo) axial images (voxel size 0.7 × 0.6 × 3.0 mm^3^) were used to quantify the gadolinium-enhancing lesions (Gad+). Apart from the quantitative imaging, a trained neuroradiologist and a trained neurologist assessed the brain MRI, comparing with the previous images to define the presence of new T2 and Gad+ lesions (Andorra et al., 2018). The T2 lesion volume and volumetric analysis of the same cohort are described in detail elsewhere (Andorra et al., 2018).

### 2.4 Fractal geometry analysis of brain MRI

**Fig. 1:**
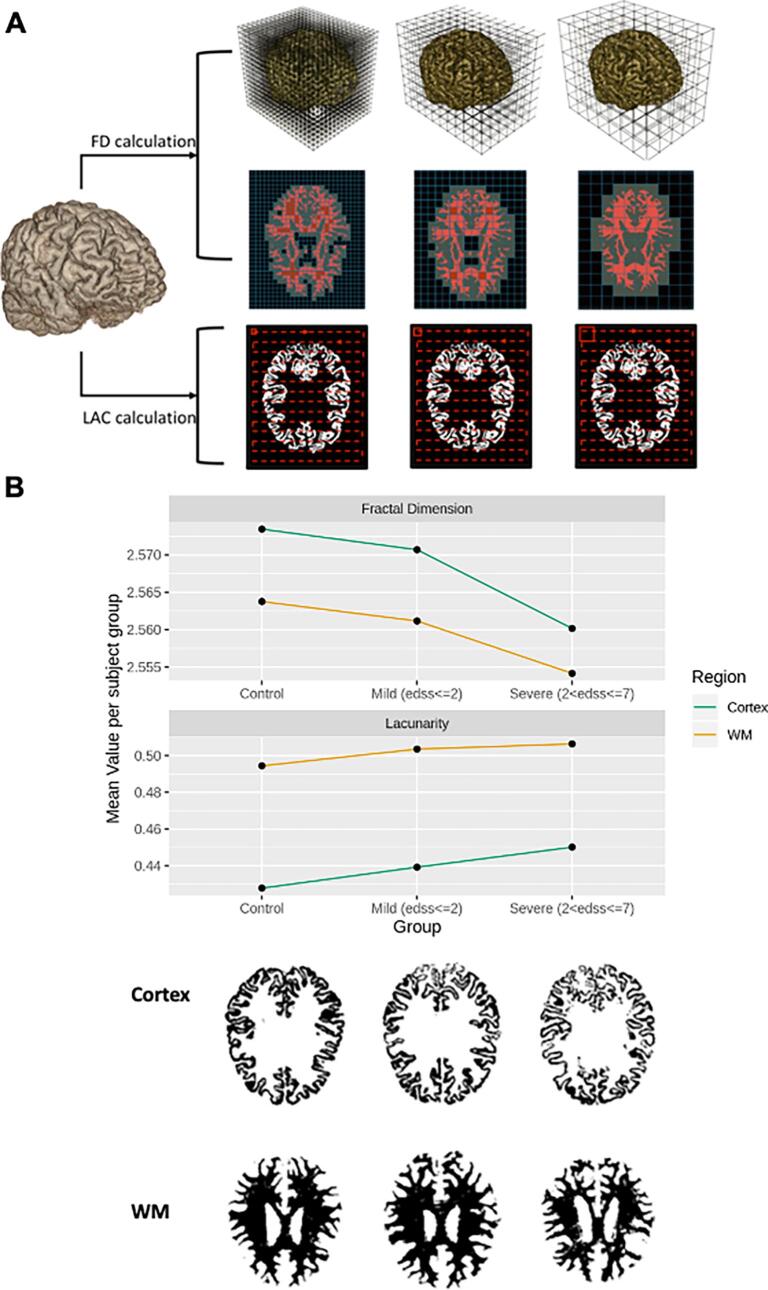
Fractal geometry of the brain. A) Brain MRI images from T1 3D scans are segmented into the different structures (cortex, grey, and white matter), and the lesions are masked. The fractal dimension (FD) and lacunarity (LAC) is calculated using the box-counting method, applying boxes of different size (by counting how many voxels the brain shape is filled at each size); B) Fractal dimension of the various brain structures decreases as tissue damage augments (figure shows the comparison between patients below and above EDSS 2.0), representing an indicator of tissue damage and roughness. Similarly, lacunarity increases along with disability as an indicator of higher empty spaces in the CNS tissue: upper graphs show the comparison of either the fractal dimension or lacunarity of the cortex with brain damage (healthy controls, and MS cases with EDSS below or above EDSS 2.0); the lower figures show representative segmented cortical or white matter regions for each subgroup (controls, MS with EDSS ≤ 2.0 and MS with EDSS > 2.0).

The fractal dimension and lacunarity were calculated on the T1 images segmented in the standard space with the box-counting method (Fig. 1) (Zhang et al., 2006), as explained in detail elsewhere (Marzi et al., 2018, Goni et al., 2013). We calculated the tertile distributions of fractal geometry variables (Table S1). We previously showed the stability of the fractal dimension estimated from repeated-acquisition T1 images both at cross-sectional and longitudinal level (Krohn et al., 2019). Because in this study, we have used 3D T1 images, the value of the fractal dimension of these images always lies between 2 (2D) and 3 (3D), the most significant differences falling between the second and third decimal within the same region of interest.

### 2.5 Statistical analysis

All the statistical analyses were carried out using the R software. The normal distribution of the variables was tested with the Shapiro-Wilks test. We described the baseline features of the study population and the distribution of events (CDA) using absolute and relative frequencies for the categorical variables. For quantitative variables, we used the medians and P25 and P75 to describe the baseline features of the study population. T-tests, ANOVA, and pairwise comparisons were performed to compare the groups as required.

We used a mixed-effects regression model to assess the rate of change in the brain fractal geometry, accounting for the intraparticipant correlation with age as the main fixed effect and the individual as the random effect. The threshold for statistical significance was fixed at p < 0.05. No imputation strategies were employed, and missing data was omitted, assuming it to be missing at random. The percentage of missing data for independent variables did not exceed 7% in any of the datasets at each visit.

Survival analysis was performed using the Cox Proportional Hazard regression analysis. Survival events were defined as the time to the first event, as defined by the disability scales, and confirmed in two consecutive visits 6 months apart (6-month CDA). The dependent variables were the fractal dimension and lacunarity of the whole brain (WB), cortex, GM, DGM, and WM. Independent variables were tested in the univariate analysis (sex, age, disease duration, MRI variables (T2 lesion volume, number of T2 lesions, cortical, GM, DGM, and WM volume) and the significant ones were included in the multivariate analysis. T2LV and number of T2 lesions were excluded for NEDA analysis because this outcome is based on the presence of new T2 lesions. We used proportional hazards models to assess the univariate effect of each of the baseline features on the risk of disability worsening. Considering that fractal dimension changes happen after the fourth decimal, hazard ratios were calculated as the rate of CDA per 10,000 unit change of fractal dimension. We evaluated time to first disability worsening event. Therefore, patients with disability worsening over the study were censored. We used the likelihood ratio test and Harrell’s C statistic to evaluate the goodness of fit of the proportional hazard models. Kaplan-Meier plots were used to show the survival curves as cumulative events. The significance of CDA events in each scale was calculated using the Wald test for a Cox analysis. Two-tailed p values of less than 0.05 were deemed significant.

### 2.6 Data availability

that anonymized data will be shared by request from any qualified investigator.

## 3 Results

### 3.1 Clinical characteristics of the cohort

The cohort was composed of 146 MS patients of the relapsing-remitting subtype with a disease duration < 10 years and mild to moderate disability (median EDSS 1.5) (Table 1). The control group was composed of 34 healthy volunteers matched to the MS cohort by sex (18 women, 53%) and age (median age 33.5, IQR 26.2–42.5 years). After a 5 year follow-up (median follow-up 4.76 years, range 2.84–5.02), and with a follow-up of 364 [351–379] days for annual visits, 2 relapsing-remitting cases converted to a secondary progressive disease, 36 patients fulfill NEDA, 25 (17%) patients had confirmed disability accumulation (CDA) for the EDSS, 17 (11%) had CDA based on the 9HPT for the dominant hand, and 7 patients for the non-dominant hand, 30 (20%) had CDA based on the T25WT, 61 (42%) had CDA based on SL25, 55 (38%) had CDA based on SDMT and 104 (71%) had CDA based in the MSFC-4. By the end of the 5 years follow-up, patients had a mean increase in the EDSS of 0.384 (Table S2). T2 lesion volume and the volume of the cortex, grey matter and white matter, values at baseline for MS cases are shown in Table 1, whereas the comparison between patients and controls at baseline and at each visit is shown in Tables S3 and S4.

**Table 1:** Clinical characteristics of the MS cohort at baseline. The data are shown as the mean and standard deviation (SD), except for the EDSS, which is also shown as the median and interquartile range (IQR).

Clinical Characteristics	MS (n = 146)
Sex (female/male)	103 / 43
Age (years)	40.1 (9.19)
Disease duration (years)	8.12 (6.89)
Subtype	
RRMS	140
CIS	6
Disease modifying therapies^1^ (Y/N)	117/29
ARMSS	3.03 (1.58)
MSSS	2.87 (1.62)
EDSS (median (IQR)) (mean (SD))	1.5 (1–2) 1.63 (0.87)
MSFC (z score)	0.25 (0.45)
9HPT dominant hand (sec)	20.3 (3.13)
9HPT non-dominant hand (sec)	21.8 (3.13)
T25WT (sec)	4.58 (1.72)
SDMT (# symbols)	54.2 (13.6)
SL25 (# letters)	24.1 (11.5)
Cortex volume (mm3)	613.56 (51.02)
GM volume (mm3)	691.27 (56.21)
WM volume (mm3)	576.74 (41.24)
T2LV (mm3)	8.74 (9.56)

1 Disease modifying drugs usage: fingolimod: 3; glatiramer acetate: 28; interferon beta: 68; natalizumab: 10; teriflunomide: 3; dimethyl-fumarate: 5.

### 3.2 Fractal geometry analysis of brain MRIs

**Fig. 2:**
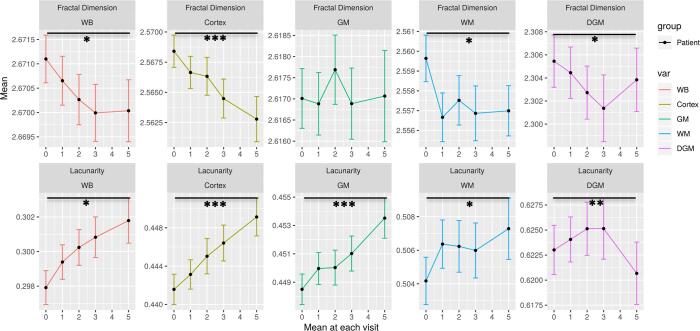
Longitudinal changes in the fractal geometry of the brain in patients with MS. The longitudinal changes during the 5-year follow-up are shown as the mean and standard error. A time-series analysis was done with a repeated ANOVA test. WB, whole-brain; GM, grey matter; WM, white matter; DGM, deep grey matter: *p < 0.01; **p < 0.001; ***p < 0.0001.

We computed the fractal geometry of the brain structures to assess whether it differed significantly between MS patients and controls. As a result, the whole brain (WB), cortical and deep grey matter (DGM) fractal dimension, and the whole brain (WB), cortical and white matter (WM) lacunarity were seen to differ significantly between the patients and controls at baseline (Table 2). Moreover, the longitudinal assessment over the 5-year follow-up showed a significant decrease in the fractal brain dimension and an increase in lacunarity (Fig. 2 and Table S4).

**Table 2:** Comparison of fractal geometry between MS patients and controls at baseline. The class comparison was achieved with a T-test for independent samples. Fractal geometry measurements are unitless.

	MS		Healthy		p-value
Measurement	Mean	SD	Mean	SD	
Fractal dimension whole brain	2.670	0.006	2.675	0.005	0.000769
Fractal dimension cortex	2.568	0.017	2.573	0.020	0.014
Fractal dimension grey matter	2.617	0.009	2.617	0.011	0.948
Fractal dimension white matter	2.560	0.015	2.564	0.014	<0.0001
Lacunarity whole brain	0.298	0.012	0.287	0.009	<0.0001
Lacunarity cortex	0.442	0.020	0.428	0.022	<0.0001
Lacunarity grey matter	0.448	0.014	0.443	0.016	0.003
Lacunarity white matter	0.504	0.018	0.494	0.016	<0.0001

**Fig. 3:**
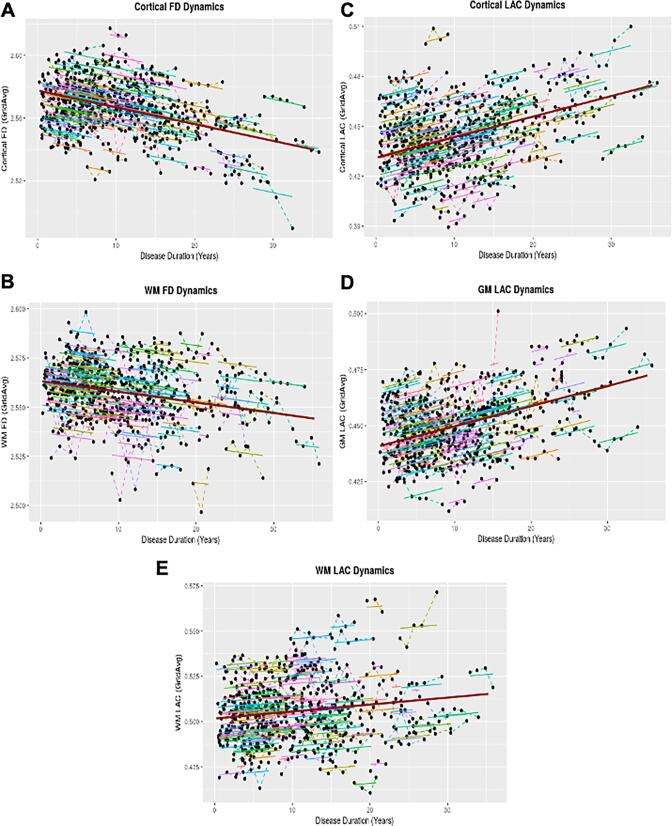
Dynamics of brain fractal geometry in Multiple Sclerosis patients during disease progression. The effect of disease duration on the fractal geometry of several brain structures was modeled using linear mixed-effects models, with age as fixed effects and individuals as the random effect. Colored points joined by a line represent the individual trajectories of FD changes, the thicker curves represent the individual fit of the model, and the dark red line represents the population model. A) cortical fractal dimension (FD); B) White matter (WM) fractal dimension; C) cortical lacunarity (LAC); D) Grey matter (GM) lacunarity; E) white matter lacunarity. (For interpretation of the references to colour in this figure legend, the reader is referred to the web version of this article.)

To assess the changes in brain fractal geometry along with disease duration, we performed linear mixed models that fitted the distribution of the annual changes in the fractal dimension and lacunarity of the brain regions studied. The models showed significant changes in the cortical and WM fractal dimension and for cortical, GM, and WM lacunarity in association with the disease duration (Fig. 3 and Table S5). We found a decline in the cortical fractal dimension of 6.546 × 10^−4^ units/year (p < 0.001), a decline of WM fractal dimension of 8.078 × 10^−4^ units/year (p < 0.0001), and a decline in the model for the GM fractal dimension of 1.601 × 10^−4^ units/year (p < 0.001). Regarding brain lacunarity, we found a significant increase in cortical lacunarity at a rate of 7.356 × 10^−3^ units/year (p < 0.001), an increase of GM lacunarity of 5.328 × 10^−4^ units/year (p < 0.001) and an increase of WM lacunarity of 6.564 × 10^−4^ units/year (p < 0.001).

### 3.3 Risk of disability accumulation based on the analysis of the fractal dimension

**Fig. 4:**
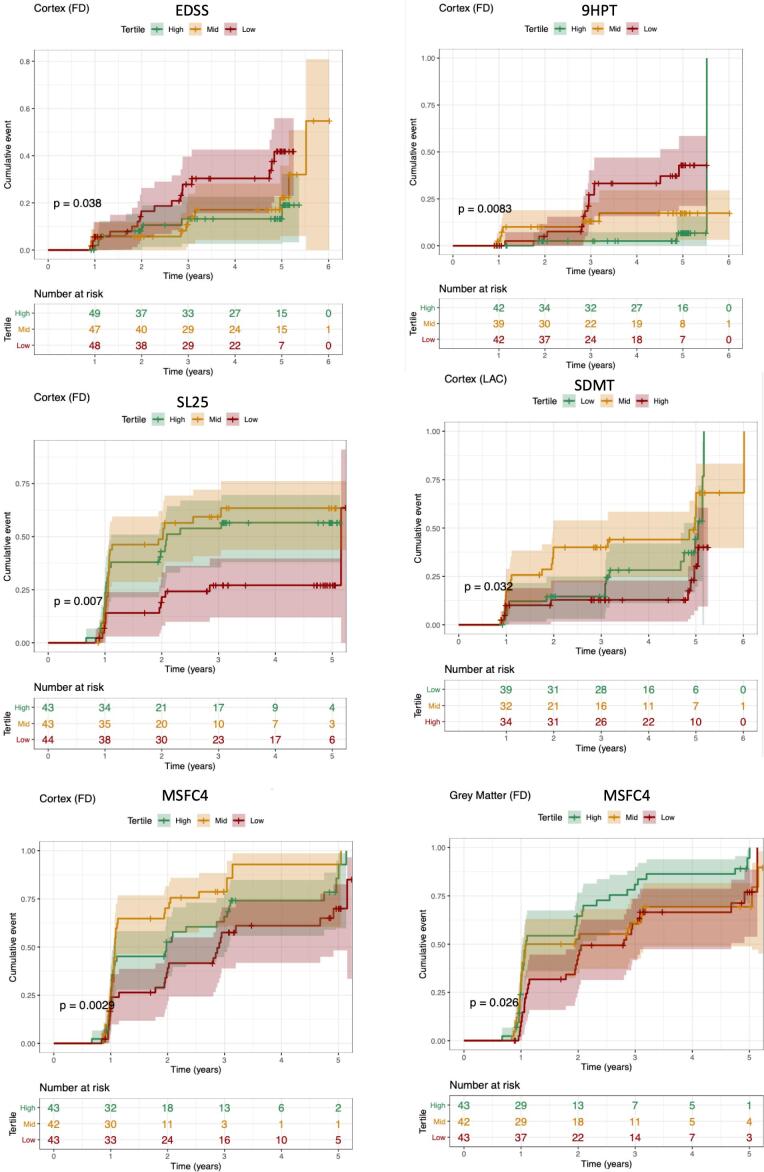
The risk (cumulative probability) of confirmed disability progression based on cortical or grey matter fractal geometry cut-offs. Survival analyses were done using Kaplan Meyer curves, and the significance is reported as the p-value of the log-rank test. Fractal dimension variables were divided in three groups using the tertiles of cortical fractal dimension – high > 2.58, medium 2.58–2.56, low < 2.56; and Grey matter (GM) fractal dimension – high > 2.622, medium 2.622–2.616, low < 2.616.

The dataset was composed of 558 observations over time obtained from 146 MS patients (range: 217 to 2,197 days; 5 visits and up to 5 years follow-up). We found that the significant variables in the univariate analysis were fractal geometry variables and age, and consequently the multivariate analysis was adjusted by age. Regarding the primary outcome, the CDA based on the EDSS, the Cox analysis showed a lower risk of EDSS worsening with higher cortical fractal dimension [HR 0.9734, CI 0.8420–0.9125; Harrell C 0.59; Wald p 0.038] (Fig. 4).

Concerning the secondary outcomes, we found significant Cox models for the 9HPT, SL25, SDMT and MSFC-4 (Fig. 4). The Cox model for CDA of the 9HPT was significant for cortical fractal dimension [HR 0.9734, CI 0.8420–0.9125; Harrell C 0.59; Wald p 0.0083]. The Cox model of CDA for SL25 was significant for cortical fractal dimension [HR 0.4311, CI 0.2035–0.9133; Harrell C 0.58; Wald p 0.0403] cortical lacunarity [HR 0.497, CI 0.2554–0.9697; Harrell C 0.58; Wald p 0.028] and grey matter lacunarity [HR 0.4445, CI 0.2177–0.9075; Harrell C 0.61; Wald p 0.0260]. Indeed, Cox model for SDMT were significant for cortex lacunarity [HR 2.215, CI 1.043–4.705; Harrell C 0.65; Wald p 0.0384]. Finally, the Cox model for CDA of the MSFC-4 was significant for cortical fractal dimension [HR 0.55, CI 0.317–0.955; Harrell C 0.59; Wald p 0.0029], and grey matter fractal dimension [HR 0.532, CI 0.326–0.868; Harrell C 0.57; Wald p 0.026]. Finally, we found no significant models for CDA based on remaining as NEDA by year 5.

### 3.4 Sensitivity analysis

Considering the relationship between brain volume or presence of lesions with disability worsening, we conducted the multivariate analysis by adjusting by T2 lesion volume and the volume of the cortex and grey matter (even if they were not significant in the univariate analysis). The Cox analysis showed for higher risk of EDSS worsening for cortical fractal dimension when adjusting for T2LV [HR 0.9978, CI 0.9956–0.9999; Harrell C 0.59; Wald p 0.0411] and a trend for higher risk of EDSS worsening when adjusting for GM volume [HR 0.9975, CI 0.9948–1.000; Harrell C 0.60; Wald p 0.0651].

## 4 Discussion

In this study, we have shown that the cortical fractal dimension identifies a subset of patients with brain damage at a higher risk of disability progression in short to mid-term. By comparing the risk based on the cortical and GM fractal geometry, we found cut-offs that are associated with a higher risk of disability accumulation on several disability scales.

Prognostic biomarkers can help to assess the risk of increasing disability in the short or long-term, and whether an individual would benefit from a higher efficacy therapy and, consequently, accept the associated risks (Pulido-Valdeolivas et al., 2017). These fractal dimension based biomarkers are intended to complement currently validated biomarkers of MS prognosis, such as T2 lesion volume or grey matter, white matter, spinal cord, or thalamic volumes (Sastre-Garriga et al., 2020). To achieve greater accuracy and power in predicting future disability, a combination of multimodal biomarkers can be pursued using composite scores (Uher et al., 2017, von Gumberz et al., 2016) or machine-learning approaches (Bejarano et al., 2011). Future studies combining fractal geometry analysis with other validated MRI markers would probably reveal better prognostic biomarkers for MS.

The fractal dimension is a measure of the topological complexity of objects, which describes their “roughness” based on the self-similarity principle (Mandelbrot, 1982). The more irregular an object is, the higher its fractal dimension value, providing a quantitative index of the coarseness of natural objects. Due to the logarithmic calculation of fractal dimension, small changes (second to third decimal) in the fractal dimension correspond to substantial differences in the shape of the object. Another fractal parameter is lacunarity, a measure of “gappiness” of an object. While the fractal dimension measures how much space is filled, lacunarity complements the fractal dimension value by measuring how the object fills the space (Di Ieva et al., 2014). Although the brain (either the cortex or the grey-white matter interface) is not a perfect mathematically fractal structure, principally because it is not strictly self-similar, analyzing the fractal dimension of the brain can still be useful to quantify cortical and subcortical morphological complexity, i.e.: ‘the roughness and gappiness of the brain’ (Di Ieva et al., 2015). The fractal dimension of brain MRI images can highlight changes in several neurological diseases, such as Alzheimer's disease, amyotrophic lateral sclerosis, or epilepsy (King et al., 2010, Rajagopalan et al., 2013, Lin et al., 2007). We attribute this sensitivity to the nature of the fractal geometry, where the site and shape of tissue damage has an essential influence on the final measurement. Therefore, adding this extra information to the proposed descriptor allows subtle changes in the brain to be captured, which would otherwise remain invisible to the human eye. Indeed, the brain’s fractal dimension is less influenced by scanner and sequence variability (Di Ieva et al., 2015, Krohn et al., 2019), and for this reason, it can complement other imaging markers, such as thalamic or spinal cord volume.

Fractal geometry provides a different information than texture analysis. Texture analysis of objects are used to describe complex patterns that describes uniformity, density, linearity, roughness among other properties in tissues like the brain (Maani et al., 2015, Nedelec et al., 2004). In a way, this could be confused by the shape complexity measured by the fractal geometry such as fractal dimension or lacunarity. Texture could be used to segment specific tissues with a given pattern and characteristics whereas fractal geometry would give a statistical metric over the desired tissue. Generally speaking, the features of certain entities are sometimes better defined by its content than by its shape and vice versa. Quantification of image texture has been successfully used in many fields, including in brain damage. In medical imaging, dissemination of healthy brain tissues from tumors and edema has been tackled by texture analysis approaches. However, the complexity of a tumor’s shape is rather weak to be unique by its own, i.e., the same shape could be drawn within a healthy tissue if one neglect the intensity or contrast of the region of interest. Instead, it has been demonstrated that tumors and brain lesions exhibit particular intensity patterns (Zhang et al., 2019). The fractal geometry is a complementary measure that can add extra information to a predefined region of interest that has been previously delineated. Ultimately, this sort of metrics could be used to improve the accuracy of segmentation approaches, adding extra features to the texture.

Our study has some limitations, not least that the cohort consisted of patients with RRMS, with little disability and intermediate disease duration. Although this dataset would not be representative of the more advanced disease and PMS, the results may be useful to patients and physicians as support to therapeutic decision-making in such an early population. Future prospective multicenter studies that include more advanced and progressive patients will be necessary to fully define the fractal geometry of the brain as a prognostic biomarker for MS. Second, the control group was only assessed at baseline. For this reason, longitudinal data is not available for these cases that might be useful to clarify the role of age-independent brain damage on fractal geometry changes. Patients were allowed to change their DMDs during the follow-up for ethical reasons, which may have also introduced variability in the follow-up. However, the effect of each immunotherapies on brain fractal geometry is unknown. In our study, the univariate analysis did not find a significant role of T2LV and brain volumes, which has been previously described as predictors of future disability. This can be due to the fact that our cohort was composed of RRMS with low to moderate severity and disease duration that displayed low changes in the volumetry and T2LV during the follow-up. For this reason, we think that such lack of significance was related with lack of events in our cohort.

In summary, the fractal dimension of brain MRIs may represent a biomarker for disease monitoring, and prognosis in patients with MS. Validation in multi-centric prospective cohorts is foreseen.

## Funding

This project has received funding from the European Union's Horizon 2020 research and innovation programme under the European Union's Horizon 2020 research and innovation programme under grant agreement No 733161, an unrestricted grant from Health Engineering SL, the Instituto de Salud Carlos III, Spain, and Fondo Europeo de Desarrollo Regional (FEDER - PI15/0061) and the CERCA Program of the Generalitat de Catalunya to PV. Magi Andorra was supported by the predoctoral fellowship for health research of the Instituto de Salud Carlos III (Spain) and Fondo Europeo de Desarrollo Regional (FEDER - FI16/00168).

## Declaration of Competing Interest

Eloy Roura, Gregory McClair, and Ferran Juanals were employees or were hired by Health Engineering SL. Magi Andorra holds equity shares in Bionure, SL, and Goodgut SL. Irene Pulido-Valdeolivas has received travel reimbursement from Roche Spain, Novartis, and Genzyme-Sanofi, and she is the founder and holds stocks in Aura Robotics SL. María Sepulveda has received speaker honoraria from Genzyme, Novartis, and Biogen. Yolanda Blanco has received speaking honoraria from Biogen, Novartis, and Genzyme. Albert Saiz has received compensation for consulting services and speaker honoraria from Bayer-Schering, Merck-Serono, Biogen-Idec, Sanofi-Aventis, TEVA, Novartis and Roche. Sara Llufriu has received compensation for consulting services and speaker honoraria from Biogen Idec, Novartis, TEVA, Genzyme, Sanofi and Merck in the last two years. Elena H Martinez-Lapiscina is employed by the European Medicines Agency (Human Medicines) since 16 April 2019. This article is related to her activity under the Hospital Clinic of Barcelona / IDIBAPS affiliation, and consequently, as external activity, it does not represent the views of the Agency, its Committees, or working parties. Pablo Villoslada holds stocks or stock-options and has received consultancy fees from Health Engineering SL, Accure Therapeutics SL, QMENTA Inc, Attune Neurosciences, CLight Inc, NeuroPrex Inc, and Spiral Therapeutics Inc
