# Supplementary material for: Cortical fractal dimension predicts disability worsening in Multiple Sclerosis patients
Source: Neuroimage Clin. 2021 Mar 29;30:102653. doi: 10.1016/j.nicl.2021.102653 (PMC8045041; doi:10.1016/j.nicl.2021.102653)
Supplement: Supplementary data 1 [file mmc1.docx]

**Supplementary material**

**Table S1. Tertile divisions of the basal FD and LAC measures.**

For fractal dimension, the first tercile is the highest. For lacunarity, the first tercile is the lowest.

| **Tertiles** | **First tertile** | **Second tertile** | **Third tertile** |
| --- | --- | --- | --- |
| **Cortex fractal dimension** | 2.576 - 2.617 | 2.562 - 2.576 | 2.519 - 2.562 |
| **GM fractal dimension** | 2.621 - 2.639 | 2.614 - 2.621 | 2.585 - 2.614 |
| **WM fractal dimension** | 2.567 - 2.593 | 2.554 - 2.567 | 2.511 - 2.554 |
| **Cortex Lacunarity** | 0.389 - 0.432 | 0.432 - 0.449 | 0.449 - 0.499 |
| **GM Lacunarity** | 0.412 - 0.442 | 0.442 - 0.454 | 0.454 - 0.488 |
| **WM Lacunarity** | 0.466 - 0.496 | 0.496 - 0.510 | 0.510 - 0.567 |

GM: grey matter; WM: white matter

**Table S2. Changes (delta) of the EDSS from baseline to year 5 of follow-up**

| **ΔEDSS** | **number cases** | **%** |
| --- | --- | --- |
| **-0.5** | 6 | 3.73% |
| **0** | 39 | 24.22% |
| **0.5** | 20 | 12.42% |
| **1.0** | 10 | 6.21% |
| **1.5** | 6 | 3.73% |
| 2.0 | 3 | 1.86% |
| **2.5** | 1 | 0.63% |
| **# patients not having**  **reached year 5 follow-up** | 76 | 47.20% |
| **Total** | **161** | **100%** |

**Table S3. Comparison of fractal geometry and volumetry between MS patients and controls at baseline**

Class comparison was done with the T test for independent samples.

|  | **MS** | | **Healthy** | | **p-value** |
| --- | --- | --- | --- | --- | --- |
| **Measurement** | **Mean** | **SD** | **Mean** | **SD** |  |
| **Cortex volume (mm3)** | 613.562 | 51.020 | 652.670 | 57.227 | 0.000611 |
| **GM volume (mm3)** | 691.271 | 56.214 | 737.103 | 61.318 | 0.000219 |
| **WM volume (mm3)** | 576.746 | 41.243 | 599.900 | 40.461 | 0.00400 |
| **T2LV (mm3)** | 8.745 | 9.561 | NA | NA | NA |
|  |  |  |  |  |  |
| **Fractal dimension Cortex** | 2.568 | 0.017 | 2.573 | 0.020 | 0.185 |
| **Fractal dimension GM** | 2.617 | 0.009 | 2.617 | 0.011 | 0.851 |
| **Fractal dimension WM** | 2.560 | 0.015 | 2.564 | 0.014 | 0.133 |
| **Lacunarity Cortex** | 0.442 | 0.020 | 0.428 | 0.022 | 0.00163 |
| **Lacunarity GM** | 0.448 | 0.014 | 0.443 | 0.016 | 0.0582 |
| **Lacunarity WM** | 0.504 | 0.018 | 0.494 | 0.016 | 0.00310 |

GM: grey matter; WM: white matter; T2LV: T2 lesion volume

**Table S4. Changes in MRI variables and fractal geometry metrics along the 5 years in patients with MS**

Differences along time points for each variable was assessed with the ANOVA test of repeated measurements. Differences for fractal geometry variables between visits respect to the baseline were analyzed with the T test

|  | **Baseline** | | **Year 1** | | **Year 2** | | **Year 3** | | **Year 5** | | **ANOVA** |
| --- | --- | --- | --- | --- | --- | --- | --- | --- | --- | --- | --- |
| **Volumetry** | **Mean** | **SD** | **Mean** | **SD** | **Mean** | **SD** | **Mean** | **SD** | **Mean** | **SD** | **p-value** |
| **Cortex volume** | 613.562 | 51.020 | 610.071 | 46.410 | 605.947 | 49.180 | 600.798 | 47.680 | 596.060 | 45.064 | **<0.0001** |
| **GM volume** | 691.271 | 56.214 | 687.294 | 51.223 | 682.668 | 53.801 | 677.561 | 52.289 | 671.906 | 49.657 | **<0.0001** |
| **WM volume** | 576.746 | 41.243 | 572.489 | 39.266 | 571.690 | 38.975 | 571.343 | 40.818 | 570.194 | 38.465 | **0.011** |
| **T2LV** | 8.745 | 9.561 | 10.068 | 10.445 | 11.151 | 11.355 | 12.285 | 12.384 | 13.080 | 13.888 | **0.016** |
| **Fractal Geometry** | | | | | | | | | | | |
| **FD Cortex** | 2.57 | 0.0169 | 2.57 | 0.0168 | 2.57 | 0.0176 | 2.56 | 0.0173 | 2.56 | 0.0182 | **<0.0001** |
| **FD WB** | 2.67 | 0.00622 | 2.67 | 0.00633 | 2.67 | 0.00596 | 2.67 | 0.00633 | 2.67 | 0.00616 | **0.006** |
| **FD GM** | 2.62 | 0.00896 | 2.62 | 0.00894 | 2.62 | 0.00939 | 2.62 | 0.00917 | 2.62 | 0.0106 | 0.2 |
| **FD WM** | 2.56 | 0.0149 | 2.56 | 0.0157 | 2.56 | 0.0141 | 2.56 | 0.0151 | 2.56 | 0.0125 | **0.01** |
| **FD DGM** | 2.31 | 0.0287 | 2.3 | 0.0286 | 2.3 | 0.0262 | 2.3 | 0.0317 | 2.3 | 0.026 | **0.003** |
| **LAC Cortex** | 0.442 | 0.0202 | 0.443 | 0.0192 | 0.445 | 0.0207 | 0.446 | 0.0201 | 0.449 | 0.0185 | **<0.0001** |
| **LAC WB** | 0.298 | 0.0124 | 0.3 | 0.0125 | 0.3 | 0.0118 | 0.301 | 0.0125 | 0.302 | 0.0126 | **0.011** |
| **LAC GM** | 0.448 | 0.0137 | 0.45 | 0.0136 | 0.45 | 0.0137 | 0.451 | 0.0131 | 0.454 | 0.0138 | **<0.0001** |
| **LAC WM** | 0.504 | 0.0179 | 0.507 | 0.0181 | 0.506 | 0.0176 | 0.506 | 0.018 | 0.508 | 0.0175 | **0.03** |
| **LAC DGM** | 0.623 | 0.0312 | 0.624 | 0.0286 | 0.625 | 0.0305 | 0.625 | 0.0334 | 0.621 | 0.0301 | **0.001** |

GM: grey matter; WM: white matter; WB: whole brain; T2LV: T2 lesion volume; FD: Fractal dimension; LAC: Lacunarity

**Table S5. Mixed-effects models regression fixed effects coefficients and random effects variability.** Results are shown as fixed or random effects, with the corresponding beta (fix) or SD (random) value respectively, the standard error and the confidence interval (CI) and p value.

**I. All cases**

| **Effect** | **Group** | **Term** | **Beta/SD** | **Std. Error** | **CI** | **P-value** |
| --- | --- | --- | --- | --- | --- | --- |
| Fractal dimension Cortex | | | | | | |
| Fixed | NA | (Intercept) | 2.6045 | 0.004716 | 2.595-2.614 | <0.0001 |
| Fixed | NA | DD (yr.) | -0.000838 | 0.0001098 | -0.001- -0.001 | <0.0001 |
| Fixed | NA | Age at inclusion (yr.) | -0.000711 | 0.0001172 | -0.001-0.000 | <0.0001 |
| Random | Subject | Std. Dev. (Intercept) | 0.013225 | NA | NA | NA |
| Random | Residual | Std. Dev. (Observation) | 0.005577 | NA | NA | NA |
| Fractal dimension WM | | | | | | |
| Fixed | NA | (Intercept) | 2.5632 | 0.001531 | 2.560-2.566 | <0.0001 |
| Fixed | NA | DD (yr.) | -0.000541 | 0.000105 | -0.001-0.000 | <0.0001 |
| Random | Subject | Std. Dev. (Intercept) | 0.0132795 | NA | NA | NA |
| Random | Residual | Std. Dev. (Observation) | 0.005650 | NA | NA | NA |
| Lacunarity Cortex | | | | | | |
| Fixed | NA | (Intercept) | 0.40185 | 0.005707 | 0.391-0.413 | <0.0001 |
| Fixed | NA | DD (yr.) | 0.001013 | 0.000117 | 0.001-0.001 | <0.0001 |
| Fixed | NA | Age at inclusion (yr.) | 0.000717 | 0.000142 | 0.000-0.001 | <0.0001 |
| Fixed | NA | Sex (Male) | 0.006564 | 0.002877 | 0.001-0.012 | 0.02397 |
| Random | Subject | Std. Dev. (Intercept) | 0.016148 | NA | NA | NA |
| Random | Residual | Std. Dev. (Observation) | 0.005531 | NA | NA | NA |
| Lacunarity GM | | | | | | |
| Fixed | NA | (Intercept) | 0.418105 | 0.003654 | 0.411-0.425 | <0.0001 |
| Fixed | NA | DD (yr.) | 0.000656 | 0.000092 | 0.000-0.001 | <0.0001 |
| Fixed | NA | Age at inclusion (yr.) | 0.000585 | 0.000093 | 0.000-0.001 | <0.0001 |
| Fixed | NA | Sex (Male) | 0.003604 | 0.001838 | 0.000-0.007 | 0.05172 |
| Random | Subject | Std. Dev. (Intercept) | 0.010155 | NA | NA | NA |
| Random | Residual | Std. Dev. (Observation) | 0.004953 | NA | NA | NA |
| Lacunarity WM | | | | | | |
| Fixed | NA | (Intercept) | 0.501674 | 0.001748 | 0.498-0.505 | <0.0001 |
| Fixed | NA | DD (yr.) | 0.000386 | 0.000106 | 0.000-0.001 | 0.00028 |
| Random | Subject | Std. Dev. (Intercept) | 0.017041 | NA | NA | NA |
| Random | Residual | Std. Dev. (Observation) | 0.004934 | NA | NA | NA |

GM: grey matter; WM: white matter

**II. Only RRMS cases**

| **Effect** | **Group** | **Term** | **Beta/SD** | **Std. Error** | **P-value** |
| --- | --- | --- | --- | --- | --- |
| Fractal dimension Cortex | | | | | |
| Fixed | NA | (Intercept) | 2.605 | 4.728e-03 | <0.0001 |
| Fixed | NA | DD (yr.) | -7.396e-04 | 1.094e-04 | <0.0001 |
| Fixed | NA | Age at inclusion (yr.) | -7.502e-04 | 1.175e-04 | <0.0001 |
| Random | Subject | Std. Dev. (Intercept) | 0.01324 | NA | NA |
| Random | Residual | Std. Dev. (Observation) | 0.00521 | NA | NA |
| Fractal dimension GM | | | | | |
| Fixed | NA | (Intercept) | 2.619 | 9.527e-04 | <0.0001 |
| Fixed | NA | DD (yr.) | --1.748e-04 | 6.482e-05 | 0.00722 |
| Random | Subject | Std. Dev. (Intercept) | 0.008434 | NA | NA |
| Random | Residual | Std. Dev. (Observation) | 0.003221 | NA | NA |
| Fractal dimension WM | | | | | |
| Fixed | NA | (Intercept) | 2.566 | 1.530e-03 | <0.0001 |
| Fixed | NA | DD (yr.) | -8.130e-04 | 1.008e-04 | <0.0001 |
| Random | Subject | Std. Dev. (Intercept) | 0.014026 | NA | NA |
| Random | Residual | Std. Dev. (Observation) | 0.004772 | NA | NA |
| Lacunarity Cortex | | | | | |
| Fixed | NA | (Intercept) | 3.998e-01 | 5.633e-03 | <0.0001 |
| Fixed | NA | DD (yr.) | 8.001e-04 | 1.147e-04 | <0.0001 |
| Fixed | NA | Age at inclusion (yr.) | 8.191e-04 | 1.402e-04 | <0.0001 |
| Fixed | NA | Sex (Male) | 6.289e-03 | 2.837e-0 | 0.0281 |
| Random | Subject | Std. Dev. (Intercept) | 0.01593 | NA | NA |
| Random | Residual | Std. Dev. (Observation) | 0.00506 | NA | NA |
| Lacunarity GM | | | | | |
| Fixed | NA | (Intercept) | 4.170e-01 | 3.619e-03 | <0.0001 |
| Fixed | NA | DD (yr.) | 5.794e-04 | 8.943e-05 | <0.0001 |
| Fixed | NA | Age at inclusion (yr.) | 6.202e-04 | 9.155e-05 | <0.0001 |
| Fixed | NA | Sex (Male) | 3.872e-03 | 1.816e-03 | 0.05172 |
| Random | Subject | Std. Dev. (Intercept) | 1.012e-04 | NA | NA |
| Random | Residual | Std. Dev. (Observation) | 0.004474 | NA | NA |
| Lacunarity WM | | | | | |
| Fixed | NA | (Intercept) | 4.996e-01 | 1.741e-03 | <0.0001 |
| Fixed | NA | DD (yr.) | 5.981e-04 | 1.029e-04 | <0.0001 |
| Random | Subject | Std. Dev. (Intercept) | 0.017368 | NA | NA |
| Random | Residual | Std. Dev. (Observation) | 0.004426 | NA | NA |

GM: grey matter; WM: white matter
